# Characterization and Comparative Analyses of Mitochondrial Genomes in Single-Celled Eukaryotes to Shed Light on the Diversity and Evolution of Linear Molecular Architecture

**DOI:** 10.3390/ijms22052546

**Published:** 2021-03-03

**Authors:** Tengteng Zhang, Chao Li, Xue Zhang, Chundi Wang, Andrew J. Roger, Feng Gao

**Affiliations:** 1 Institute of Evolution & Marine Biodiversity and College of Fisheries, Ocean University of China, Qingdao 266003, China; tengtzhang@foxmail.com (T.Z.); lc9869@stu.ouc.edu.cn (C.L.); zhangxue3425@stu.ouc.edu.cn (X.Z.); chundiwang102@foxmail.com (C.W.); 2 Key Laboratory of Mariculture (OUC), Ministry of Education, Qingdao 266003, China; 3 Centre for Comparative Genomics and Evolutionary Bioinformatics, Department of Biochemistry and Molecular Biology, Dalhousie University, Halifax, NS B3H 4R2, Canada; andrew.roger@dal.ca; 4 Laboratory for Marine Biology and Biotechnology, Qingdao National Laboratory for Marine Science and Technology, Qingdao 266033, China

**Keywords:** ciliated protists, mitochondrial genome, phylogeny, split genes, synteny, tandem repeat

## Abstract

Determination and comparisons of complete mitochondrial genomes (mitogenomes) are important to understand the origin and evolution of mitochondria. Mitogenomes of unicellular protists are particularly informative in this regard because they are gene-rich and display high structural diversity. Ciliates are a highly diverse assemblage of protists and their mitogenomes (linear structure with high A+T content in general) were amongst the first from protists to be characterized and have provided important insights into mitogenome evolution. Here, we report novel mitogenome sequences from three representatives (*Strombidium* sp., *Strombidium* cf. *sulcatum*, and *Halteria grandinella*) in two dominant ciliate lineages. Comparative and phylogenetic analyses of newly sequenced and previously published ciliate mitogenomes were performed and revealed a number of important insights. We found that the mitogenomes of these three species are linear molecules capped with telomeric repeats that differ greatly among known species. The genomes studied here are highly syntenic, but larger in size and more gene-rich than those of other groups. They also all share an AT-rich tandem repeat region which may serve as the replication origin and modulate initiation of bidirectional transcription. More generally we identified a split version of *ccmf*, a cytochrome c maturation-related gene that might be a derived character uniting taxa in the subclasses Hypotrichia and Euplotia. Finally, our mitogenome comparisons and phylogenetic analyses support to reclassify *Halteria grandinella* from the subclass Oligotrichia to the subclass Hypotrichia. These results add to the growing literature on the unique features of ciliate mitogenomes, shedding light on the diversity and evolution of their linear molecular architecture.

## 1. Introduction

Mitochondria are double-membraned semiautonomous organelles that contain their own genomes (mitogenomes) that originated from an endosymbiotic α-proteobacterium that evolved prior to the last eukaryotic common ancestor [1,2,3]. Although they are best known for generating ATP by aerobic respiration to supply the energy needs of eukaryotic cells, these organelles perform a much wider range of functions that vary substantially among diverse eukaryotic lineages [1]. Mitochondria also have biomedical significance, as mutations affecting mitochondrial function are implicated in ageing and diseases, including Parkinson’s, Alzheimer’s, and Huntington’s diseases [4,5].

Mitogenomes usually evolve more rapidly than their nuclear counterparts in most species [6,7]. Additionally, each cell possesses multiple mitogenome copies and mitogenome recombination is a widespread process occurring in plants, fungi, protists, and animals [8,9,10]. In general, unicellular protists possess the most gene-rich and structurally varied mitogenomes [11]. Ciliates are a diverse and well-studied protistan assemblage with unusual cellular and molecular features (nuclear dimorphism and conjugation) [12,13,14,15,16,17,18,19,20,21,22,23]. Their mitogenomes were the first amongst protists to be characterized providing novel insights into the structure, function, and evolution of organellar genomes [24]. The mitogenomes of the ciliates *Tetrahymena* and *Paramecium* were the first confirmed to be linear double-stranded DNA molecules and to have their telomeric sequences identified, which provided novel insights into the replication of linear mitochondrial DNA [25,26,27].

In the years since those seminal discoveries, more ciliate mitogenomes have been characterized, including 20 mitogenomes from species in the class Oligohymenophorea (*Tetrahymena pyriformis*, *T. thermophila*, *T. malaccensis*, *T. paravorax*, *T. pigmentosa*, *T. rostrata*, *Ichthyophthirius multifiliis*, *Uronema marinum*, *Paramecium caudatum*, *P. aurelia*, *P. tetraurelia*, *P. sexaurelia*, *P. multimicronucleatum*, *P. biaurelia*, *P. octaurelia*, *P. novaurelia*, *P. decaurelia*, *P. dodecaurelia*, *P. quadecaurelia*, and *P. jenningsi*) [28,29,30,31,32,33,34,35,36], eight mitogenomes of species in the class Spirotrichea (*Oxytricha trifallax*, *Laurentiella strenua*, *Stylonychia lemnae*, *Paraurostyla* sp., *Urostyla grandis*, *Pseudourostyla cristata*, *Euplotes minuta*, and *E. crassus*) [37,38,39], two mitogenomes in the class Heterotrichea (*Stentor coeruleus* and *Gruberia lanceolata*) [20,40], as well as seven hydrogenosomal genomes of anerobic ciliates (*Nyctotherus ovalis*, *Metopus contortus*, *Metopus es*, Metopid sp., *Heterometopus* sp., *Parablepharisma* sp., and *Muranothrix gubernata*) [41,42,43]. Ciliate mitogenomes are generally in size of 20–70 kb, with high A+T content (58.53% for *N. ovalis* ~81.51% for *I. multifiliis*) and relatively large gene complements (20–30 protein-coding genes) [39,41,44]. Mitochondrial DNA has been proven extremely useful in phylogenetic analyses to determine ciliate relationships and species delimitation [45].

In this study, we report the analysis of complete mitogenomes of three ciliates: two species of the subclass Oligotrichia (*Strombidium* sp. and *Strombidium* cf. *sulcatum*) and a representative species of Halteriidae (*Halteria grandinella*) whose phylogenetic position is controversial (i.e., morphological data contradicts molecular data [46,47,48]). These species all belong to the class Spirotrichea, which is a highly differentiated and species-rich assemblage among ciliates [49]. We annotated and compared these three genomes with the previously published ciliate mitogenomes, particularly the mitogenomes among Spirotrichea, shedding light on the diversity and evolution of mitochondrial genomes within this group. Phylogenetic analysis based on 14 mitochondrial ortholog proteins of 34 ciliates was performed in order to further investigate and clarify the evolutionary relationships among Oligotrichia, Halteriidae, and Hypotrichia.

## 2. Results

### 2.1. Mitogenome Overview

**Figure 1:**
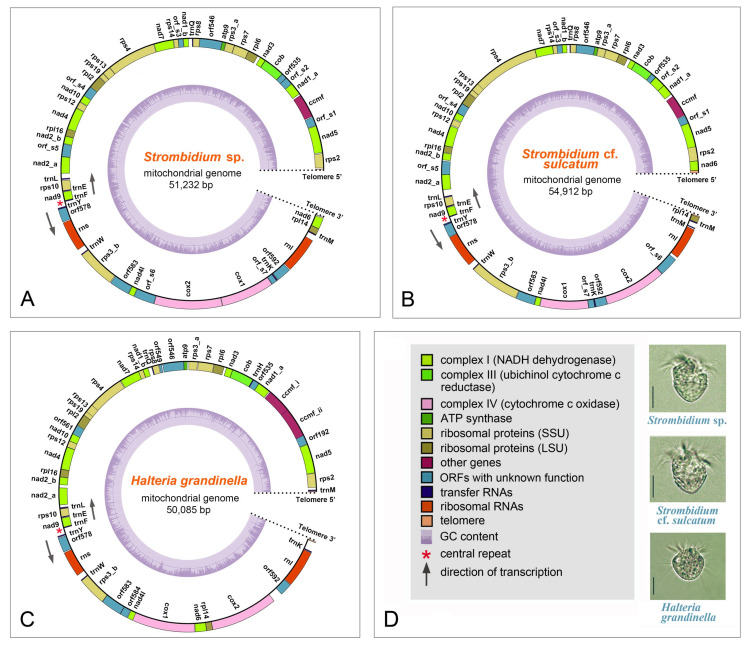
Mitochondrial and morphological information of the three species with newly characterized mitogenomes. (**A**) Mitogenome map of *Strombidium* sp. (**B**) Mitogenome map of *Strombidium* cf. *sulcatum*. (**C**) Mitogenome map of *Halteria grandinella*. (**D**) Photomicrographs of the three species in vivo and the legend of mitochondrial maps. Genes are represented by different colored blocks as indicated in the legend of diagram D. Outside and inside blocks of each mitochondrial map indicate the genes on the positive and reverse strands, respectively.

For each species, mitochondrial contigs (*Strombidium* sp.: one contig; *Halteria grandinella*: one contig; *Strombidium* cf. *sulcatum*: 9 contigs) are recovered from the assembled mitochondrial or genomic data by BLASTN searches. They are assembled into linear molecules for the mitogenomes of *Strombidium* sp., *S.* cf. *sulcatum*, and *H. grandinella* with coverage of 194.2, 492.2–564.8, and 40.2 respectively (Figures S1 and S2). The lengths of the mitogenomes are 51,232 bp for *Strombidium* sp., 54,912 bp for *S.* cf. *sulcatum*, and 50,085 bp for *H. grandinella* with the A+T contents of 77.32%, 71.68%, and 80.20%, respectively (Table 1). Furthermore, the three mitogenomes are all capped with telomeric tandem repeats at both ends, which are reverse complements of each other. The telomeric repeat sequences are divergent among the three species (18 bp in *Strombidium* sp.: CTC CCT TAT CTA GTC TTT; 34 bp in *S.* cf. *sulcatum*: TTA TAT CCT TTC TCC CCT ATA TCT CTA TAG TAC T; and 31 bp in *H. grandinella*: AAA ACA GCT CCG TTC CAA TAC TAC TAA CTA A) (Table 2). A central repeat region found in all three genomes is made up of tandem repeat units, and occurs at the same position between *tRNA_Phe* (*trnF*) and *tRNA_Tyr* (*trnY*) in each genome. However, the central repeats differ from one another in terms of length and sequence. In *Strombidium* sp., the central repeat region is 170 bp long, composed of 11 repeats of the unit ATA ATA TAA TAA TAT. This region is 142 bp long in *S.* cf. *sulcatum*, composed of two repeats of the unit ATA AAT TTA ATT TTA and an irregular sequence for the rest. In *H. grandinella*, this region is 168 bp long and composed of nine repeats of the unit TAT ACA TAT AAT ATA TA (Table 2). Diverging and starting from this central repeat region, all the genes in the three mitogenomes are arranged in two opposite transcriptional directions (Figure 1).

**Figure 2:**
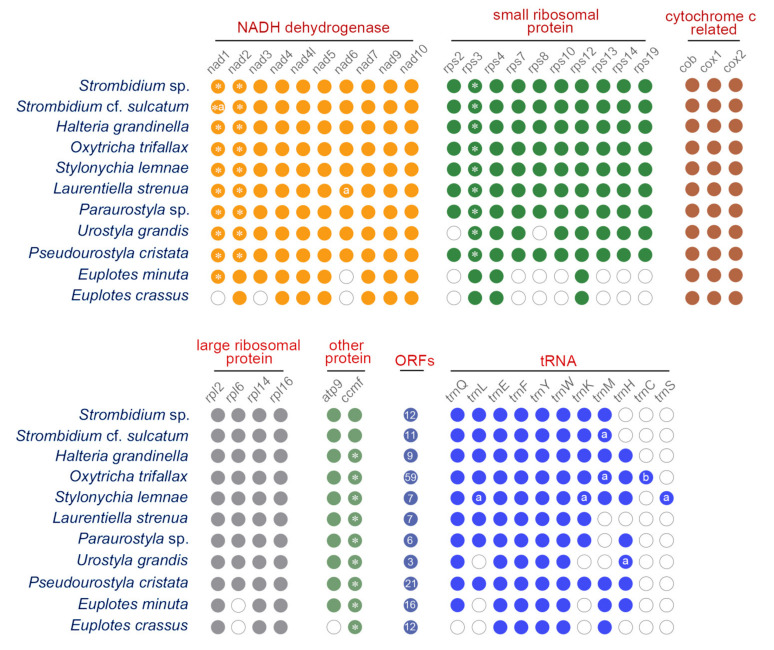
Protein and tRNA genes encoded in mitogenomes of the class Spirotrichea. The solid circles represent presence and the hollow circles represent absence. The asterisk “*” indicates a split gene that is split into two parts. The letter “a” indicates the gene is duplicated into two copies, while “b” represents that one copy of *tRNA_Cys* (*trnC*) is a pseudogene. The number indicates the number of unknown ORFs.

For *Strombidium* sp., 94.89% of the mitogenome is coding sequence, including 29 known protein genes, 8 tRNA genes, 2 rRNA genes, and 12 open reading frames (ORFs) with unknown function. The mitogenome of *S.* cf. *sulcatum* also contains 29 known protein genes, but instead has 9 tRNA genes, 2 rRNA genes, and 11 ORFs, with the coding region comprising 95.09%. The proportion of coding sequence is 90.65% in the *H. grandinella* mitogenome, the lowest among the three taxa, which comprises 29 known protein genes, 9 tRNA genes, 2 rRNA genes, and 9 ORFs (Table 1, Figure 1 and Figure 2). The mitochondrial genes of the three species are tightly packed and some genes are partially overlapping (*Strombidium* sp.: seven cases (10–46 bp, avg. 24 bp), *S.* cf. *sulcatum*: seven cases (7–82 bp, avg. 46 bp), *H. grandinella*: fifteen cases (4–190 bp, avg. 39 bp)) (Table S3, Figure 1). Three genes of *nad1*, *nad2* (NADH dehydrogenase genes), and *rps3* (small subunit ribosomal protein gene) are separated into two parts for all the three mitogenomes, while the *ccmf* (cytochrome c maturation-related gene) is split only for *H. grandinella*. Two duplicated genes of *nad1_a* and *tRNA_Met* (*trnM*) are found in *S.* cf. *sulcatum* mitogenomes (Figure 1 and Figure 2). No introns are detected in any of the three mitogenomes.

### 2.2. Mitogenome Comparison among Species in the Class Spirotrichea

**Figure 3:**
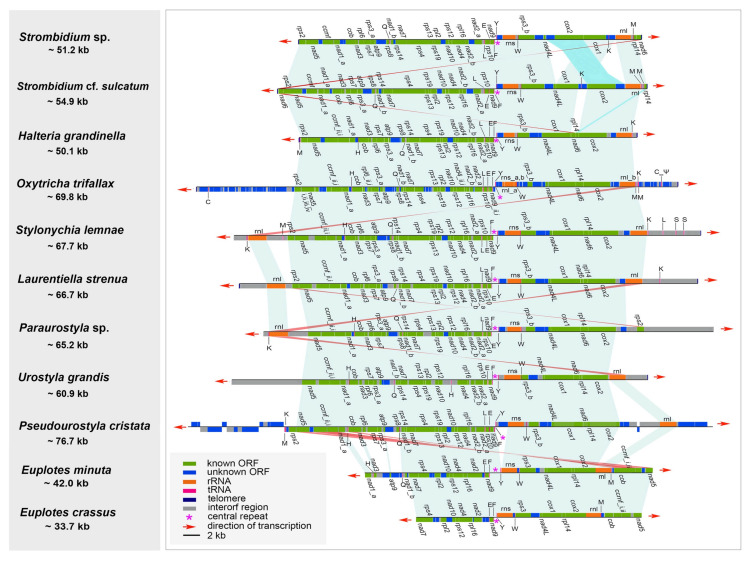
Mitogenome comparison of representatives within Spirotrichea (the subclass Oligotrichia: *Strombidium* sp., MT471315; *Strombidium* cf. *sulcatum*, MT471316; the subclass Hypotrichia: *Halteria grandinella*, MT471317; *Oxytricha trifallax*, JN383843; *Stylonychia lemnae*, KX524144; *Laurentiella strenua*, KX529838; *Paraurostyla* sp., KX524143; *Urostyla grandis*, KX494929; *Pseudourostyla cristata*, MH888186; the subclass Euplotia: *Euplotes minuta*, GQ903130; *Euplotes crassus*, GQ903130). Collinearity between the genomes is indicated by pale-green shades, while the structural rearrangements are indicated by pale-blue or pink shades. tRNA genes are represented by abbreviations of capital letters.

**Figure 4:**
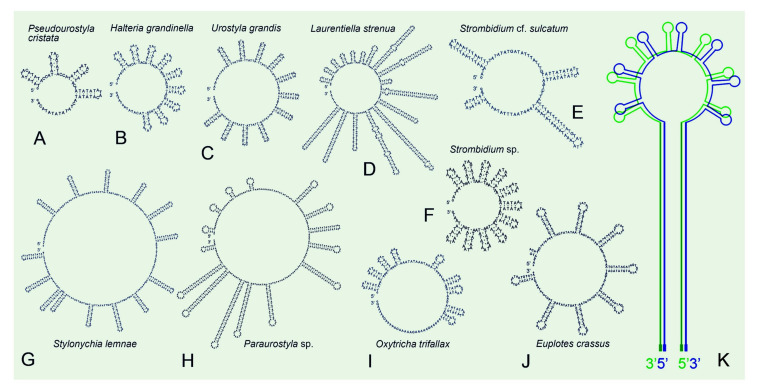
Secondary structures of central repeat regions of mitogenomes in the subclass Hypotrichia (**A**–**D**, **G**–**I**), Oligotrichia (**E**,**F**), and Euplotia (**J**) based on single-stranded DNA. The general model of mitogenomes of the class Spirotrichea (**K**) inferred from double-stranded DNA is based on the structures of **A**–**J**.

We compared the three newly sequenced mitogenomes with eight other representative mitogenomes available in the class Spirotrichea. Among these mitogenomes, only the three that were newly sequenced and those of *Oxytricha trifallax* and *Laurentiella strenua* are complete, with both ends capped by telomeres (Table 2, Figure 3). To our knowledge, *Pseudourostyla cristata* possesses the longest mitogenome (76,660 bp) among the available mitogenomes in ciliates. Within the class of Spirotrichea, the A+T content of the mitogenome in *Paraurostyla* sp. is the highest (80.59%), while that of *Urostyla grandis* is the lowest (61.12%, Table 1). It is noteworthy that all mitogenomes of Spirotrichea contain a central tandem repeat that has a high A+T content (83.36–100%, avg. 93.31%) (Table 2). Besides, the central repeats are always located between *trnF* and *trnY* (Figure 3). Strikingly, the mitochondrial central repeat regions contain potential palindrome sequences, such as TA repeats. The repeat unit sequence is identical between two species of Euplotia, while it varies among species in oligotrichs and hypotrichs (Table 2). According to the secondary structures of central repeats of species in Spirotrichea (apart from *Euplotes minuta*), the tandem repeat region shows similar stem-loop structure: the sun-shape structure containing 4–17 helices with similar length for *P. cristata*, *H. grandinella*, *U. grandis*, *O. trifallax*, *E. crassus*, *Strombidium* sp., and *Stylonychia lemnae*, but with divergent length for the remaining three species. (Figure 4A–J). Moreover, a general model of the secondary structure of mitogenomes in the class Spirotrichea is proposed based on these structures (Figure 4K).

**Figure 5:**
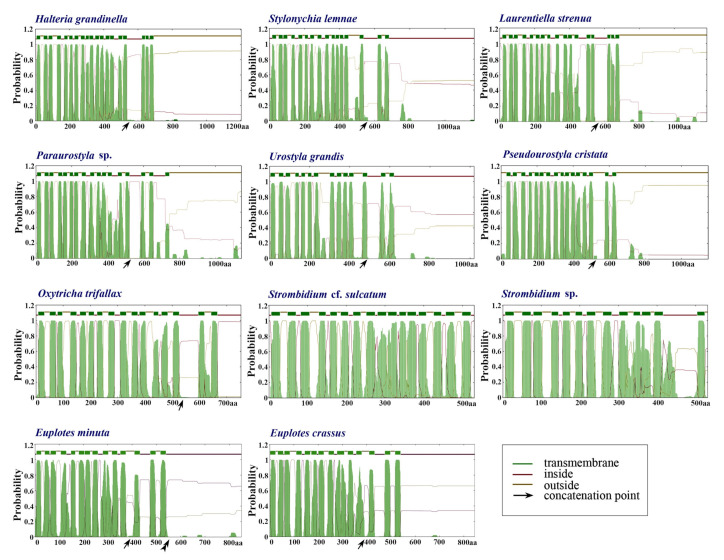
Transmembrane (TM) profiles for the concatenated ccmf proteins of eleven representative mitogenomes in the class Spirotrichea. The x and y axes denote amino acid length and posterior probabilities detected by TMHMM v.2.0, respectively. The green blocks represent TM regions. The arrow indicates concatenation point of ccmf_i and ccmf_ii. The double arrowheads denote the pseudo split of ccmf_ii in *Euplotes minuta*.

**Figure 6:**
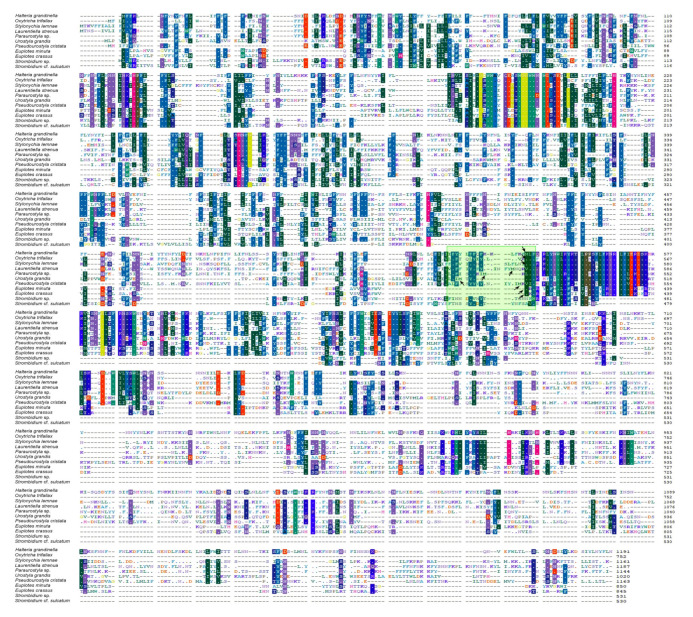
Sequence alignment of ccmf proteins of mitogenomes in Spirotrichea. Regions with substantial sequence similarity are indicated in color shade. Arrow indicates the concatenation points of ccmf_i and ccmf_ii.

The three newly characterized mitogenomes share largely the same complement of known protein-coding genes with the other mitogenomes of the class Spirotrichea (Figure 2). The two *Euplotes* species lack many mitochondrial genes, probably due to the incompleteness of the mitogenomes. The protein-coding genes include NADH dehydrogenase genes (*nad1–7*, *nad9–10*, *nad4L*), cytochrome c reductase and oxidase genes (*cob*, *cox1*, *cox2*), ATP synthase gene (*atp9*), ribosomal protein genes (*rps2–4*, *rps7–8*, *rps10*, *rps12–14*, *rps19*, *rpl2*, *rpl6*, *rpl14*, *rpl16*), and cytochrome c maturation-related gene (*ccmf*). Nevertheless, *rps2* and *rps8* are not detected in the mitogenome of *U. grandis*. In addition, the *ccmf* gene is split into *ccmf_i* and *ccmf_ii* in the mitogenomes of all Spirotrichea species apart from two oligotrichous species, which will be discussed in detail below (Figure 2, Figure 3, Figure 5 and Figure 6). According to the transmembrane profile predictions, the ccmf protein of *H. grandinella* exhibits similar transmembrane helices with those of hypotrichs and euplotids where ccmf_i contains 11–16 transmembrane structures and ccmf_ii comprises 2–3 transmembrane helices and a long non-transmembrane region (Figure 5). Duplications are detected in mitogenomes of the class Spirotrichea. For instance, *nad1_a* of *S.* cf. *sulcatum*, *nad6* of *L. strenua*, mitochondrial large subunit ribosomal RNA gene (*rnl*) of *S. lemnae* and *L. strenua*, *trnM* of *S.* cf. *sulcatum* and *O. trifallax*, *tRNA_Leu* (*trnL*), *tRNA_Lys* (*trnK*), and *tRNA_Ser* (*trnS*) of *S. lemnae*, and *tRNA_His* (*trnH*) of *U. grandis* (Figure 2 and Figure 3). Notably, *tRNA_Glu* (*trnE*), *trnF*, *trnY*, and *tRNA_Trp* (*trnW*) are present in all these eleven mitogenomes, while *trnS* is only detected in *S. lemnae* (Figure 2). Besides, a pseudogene of *tRNA_Cys* (*trnC*) is detected in the mitogenome of *O. trifallax* (Figure 2). Except for the known protein genes, some homologous ORFs with unknown functions are also detected in mitogenomes of the class Spirotrichea according to BLASTP results and gene location on the mitogenome map, such as *orf535*, *orf546*, *orf578*, *orf583*, and *orf592*. Interestingly, seven ORFs (*orf_s1–s7*) in mitogenomes of two *Strombidium* species and six ORFs (*orf259*, *orf163*, *orf187*, *orf96*, *orf111*, and *orf155*) in two *Euplotes* mitogenomes are unique and no homolog is found in mitogenomes of other species (Table S4).

Gene order comparison shows that the gene synteny is highly conserved among mitogenomes within the class Spirotrichea (Figure 3). For the two *Strombidium* species, there is collinearity for most genes, with the exceptions of two translocations (*cox2* and *orf_s6*) and one inversion (*nad6*). The mitogenomes of *S.* cf. *sulcatum* and *H. grandinella* are largely collinear, except that two terminal genes of *nad6* and *rpl14* in *S.* cf. *sulcatum* are moved to the position between *cox1* and *cox2* in *H. grandinella*. The gene orders in *H. grandinella* and *O. trifallax* mitogenomes are identical. The mitogenome synteny is also significant among the hypotrichs, except for the inversion of *rps2* in *Paraurostyla* sp. and the gene duplication of *rnl* in *S. lemnae*, *L. strenua*, and *Paraurostyla* sp. The two *Euplotes* mitogenomes are collinear and are largely collinear with those of the hypotrichs in the core region, from *nad3* to *rnl*, except for the inversion of *nad5*, *ccmf*, and *cob*. Besides, the gene order of tRNA, *tRNA_Gln* (*trnQ*)-*trnL*-*trnE*-*trnF*-*trnY*-*trnW*, is conserved in all the species except for the missing *trnQ* in *E. crassus* and *trnL* in *U. grandis*, *E. minuta*, and *E. crassus*.

Similar to the extensive collinearity within the class Spirotrichea, the mitogenomes are largely collinear within the class Oligohymenophorea (mainly represented by *Tetrahymena* and *Paramecium*), with the exception of one large inversion and translocation from *cob* to *rnl* [39]. The collinearity between the classes Spirotrichea and Oligohymenophorea is lower than within classes, and no collinearity is observed between the classes Spirotrichea and Heterotrichea, which is consistent with the higher ciliate taxonomic classification [39].

### 2.3. Phylogenetic Analyses Based on Mitochondrial Proteins

**Figure 7:**
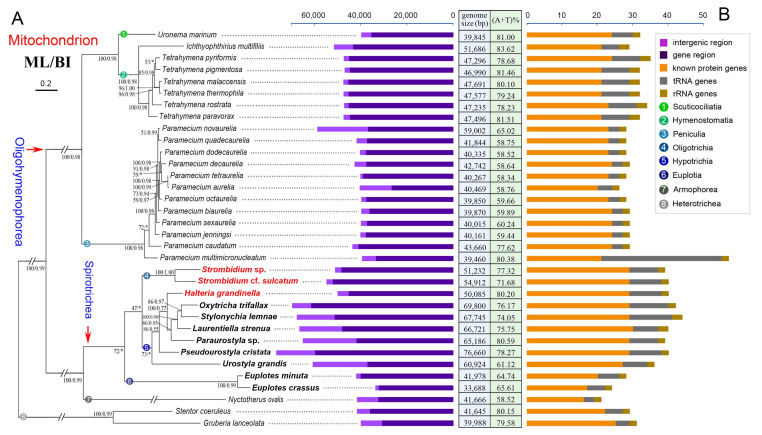
Phylogenetic analyses and main information of all the available mitogenomes in aerobic ciliates and a representative hydrogenosome genome in anaerobic ciliates. (**A**) Maximum likelihood (ML) tree based on the concatenated protein sequences of 14 mitochondrial orthologs of 34 available ciliates mitogenomes. Numbers at the nodes represent the standard nonparametric bootstrap values of ML out of 100 replicates and the posterior probability values of Bayesian analysis (BI). The asterisk “*” indicates the disagreement between ML and BI. The scale bar corresponds to 20 substitutions per 100 nucleotide positions. (**B**) Mitogenome and hydrogenosome information in 34 ciliate morphospecies. The left block indicates the length of the gene and intergenic regions, and the right block shows the number of the three kinds of genes, respectively.

The phylogenetic trees based on mitochondrial proteins reveal a concordant topology with that inferred from nuclear small subunit ribosomal DNA (nSSU-rDNA) data, thus only the topology based on mitochondrial protein with support values generated from both maximum likelihood (ML) and Bayesian inference (BI) analyses is presented (Figure 7). According to the phylogenetic trees in our work, all the classes (Spirotrichea and Oligohymenophorea) and the subclasses (Oligotrichia, Euplotia, Hypotrichia, Peniculia, and Hymenostomatia) are monophyletic. Moreover, *H. grandinella* clusters with four species of the family Oxytrichidae (*S. lemnae*, *O. trifallax*, *L. strenua*, and *Paraurostyla* sp.) based on the mitochondrial data (ML/BI: 100/0.77), which is consistent with previous studies [48,49]. According to the phylogenetic trees based on multiple nuclear gene markers (nSSU-rDNA, internal transcribed spacer (ITS), 5.8 S rDNA, nuclear large subunit ribosomal DNA (nLSU-rDNA), *α-tubulin*, and *actin I* genes), *H. grandinella* was always clustered within core hypotrichs [49,50,51,52]. Recently, phylogenomic analyses based on more than 130 orthologs also implied the close relationship between *H. grandinella* and Hypotrichia [19,48]. In our work, *H. grandinella* nests within hypotrichs, while the subclass Oligotrichia, represented by two *Strombidium* species because of a limitation of data, forms a monophyletic clade that is sister to hypotrichs (Figure 7). In order to test the possibility of previous assignment of *H. grandinella* into Oligotrichia, the constrained ML tree with two *Strombidium* species clustering with *H. grandinella* was compared with the non-constrained ML topology using the approximately unbiased (AU) test. However, the clustering of *H. grandinella* and *Strombidium* taxa is rejected on the basis of mitochondrial data in the present work (*p* = 0.0024).

The main features of the 34 mitogenomes in ciliates, including all the published mitogenomes of aerobic ciliates and a representative hydrogenosomal genome of anaerobic ciliates, are also compared and mapped on the phylogenetic trees (Figure 7). Except the incomplete assembly of two *Euplotes* mitogenomes, the mitogenomes of the species in Spirotrichea are generally both gene-rich and relatively large. For example, Spirotrichea mitogenomes are generally 50–70 kb, containing about 30 known protein genes and 7–12 tRNA genes, whereas the Oligohymenophorea mitogenomes are generally 40–50 kb, with about 20 known protein genes and 3–8 tRNA genes.

## 3. Discussion

### 3.1. Ccmf and Other Split Protein-Coding Genes in Mitogenome

The ccmf protein is very important for aerobic eukaryote mitochondria, given that it is associated with maturation of cytochrome c which binds to heme and transfers electrons between respiratory chains [53]. In the present work, all mitogenomes of Spirotrichea species contain the *ccmf* gene. According to previous studies, the *ccmf* gene is suspected to be split into two parts in the mitogenomes of *O. trifallax*, *L. strenua*, *U. grandis*, and *P. cristata* [37,39,54]. According to De Graaf et al. 2009 [38], *ccmf* could also be a split gene for *E. minuta* and *E. crassus*, but have three parts (an additional split of *ccmf_i* for *E. crassus* and an additional split of *ccmf_ii* for *E. minuta*). Nevertheless, we found that the split of *ccmf_i* for *E. crassus* results from the insertion of an adenine nucleotide based on the comparison of our mitogenomes of *E. crassus* and *E. vannus* (unpublished) with the published data. Similarly, many insertions and deletions of *ccmf_ii* of *E. minuta* were detected. Although the insertion and deletion might come from the interspecific divergence, we think the additional split of *ccmf_ii* of *E. minuta* might not be a true split but instead is the product of sequencing errors. This speculation is consistent with the length, transmembrane (TM) profile, and sequence alignment of ccmf proteins as discussed below (Figure 5 and Figure 6).

The *ccmf* genes of *H. grandinella*, *S. lemnae*, and *Paraurostyla* sp. might also be split into two parts, with the following four findings supporting this hypothesis. Firstly, for the mitogenome in each species, two adjacent ORFs align with two parts of the ccmf protein (ccmf_i and ccmf_ii, respectively) based on a prediction with the NCBI Open Reading Frame Finder and SmartBLAST, with the best BLAST hit generally coming from one of the ciliate mitogenomes. Secondly, each of the two hypothetical split parts (ccmf_i and ccmf_ii) possess similar lengths among Hypotrichia and Euplotia mitogenomes, respectively: the lengths of ccmf_i are 543 amino acids for *H. grandinella*, 480–548 (avg. 524) amino acids for Hypotrichia, and 405 amino acids for Euplotia; the lengths of ccmf_ii are 648 amino acids for *H. grandinella*, 219–645 (avg. 547) amino acids for Hypotrichia, and 440–445 (avg. 442) amino acids for Euplotia. Thirdly, ccmf is a mitochondrial integral membrane protein containing multiple transmembrane helices [55]. In our work based on TM profiles, the transmembrane helix structure of hypothetical ccmf split proteins is similar among *H. grandinella*, Hypotrichia, and Euplotia (Figure 5). Fourthly, according to the alignment result of ccmf proteins in Spirotrichea, the split sites of ccmf_i and ccmf_ii are almost identical (Figure 6). All of these suggest that the *ccmf* gene is split into two parts in the mitogenomes of *H. grandinella*, Hypotrichia, and the Euplotia species described here.

However, as for the two *Strombidium* species, the *ccmf* gene is non-split because we only find one ORF that is homologous to cytochrome c maturation-related genes (Figure 1, Figure 2 and Figure 3 and Figure 5). According to the TM profiles, the ccmf of the two *Strombidium* species possesses 13–16 transmembrane helices, which is similar to that of other spirotrichous species (14–19 transmembrane helices). Besides, the split phenomenon was not detected based on the ccmf sequence of two *Strombidium* species, nearby the conserved split positions of Hypotrichia and Euplotia ccmf proteins (Figure 6). Hence, it appears that the mitochondrial *ccmf* gene is likely split for Hypotrichia and Euplotia species but corresponds to a single polypeptide for Oligotrichia species.

According to previous studies [37,39], the protein-coding genes *nad1*, *nad2* and *rps3* were reported as split genes in most sequenced ciliate mitogenomes. These three genes are also found split in the newly characterized three mitogenomes in the present study. This split appears to be ancestral and shared by the phylum Ciliophora. The non-split of the genes *nad2* and *rps3* in *Euplotes* mitogenomes could be due to sequencing and annotation errors or they were fused again in the *Euplotes* species. By contrast, the *ccmf* gene is found split only in Hypotrichia and Euplotia species, while it is not split in Oligotrichia and Oligohymenophorea, which indicates that the split of *ccmf* gene in Hypotrichia and Euplotia species is a derived character and probably evolved independently in these two groups. Notably, the protein-coding genes *nad4*, *nad5*, *nad9*, and *rpl6* were also predicted as potential split genes in *O. trifallax* [39]. However, these genes are not split in other ciliate mitogenomes. The split of *nad4*, *nad5*, *nad9*, and *rpl6* in *O. trifallax* may be not accurate due to sequencing errors, because of the fact that this mitogenome was sequenced with error-prone 454 sequencing technology. In conclusion, the split in genes *nad1*, *nad2*, and *rps3* could be an ancestral characteristic among all ciliates. Meanwhile the split in *ccmf* is a specific feature within the subclasses Hypotrichia and Euplotia, which could be used for clarifying the phylogenetic relationships for these groups.

### 3.2. ORFs with Unknown Functions in Mitogenome

The ORFs with unknown functions have always been a problem for protist mitogenomes, and result in the trouble of annotation for mitochondrial genes. However, the high evolutionary rate of mitochondrial genes and low sequence identity between ORFs and known genes make it difficult to define these ORFs with unknown functions by BLAST searches [7,31]. Specifically, compared to known protein-coding genes, the ORFs with unknown functions have significantly higher Ka/Ks values and lower sequence similarities, suggesting a lower selective pressure on these ORFs [31,37]. Nevertheless, there are some ORFs that are homologous and reside in the same relative positions among the Spirotrichea mitogenomes (e.g., *orf535*, *orf546*, *orf578*, *orf583*, and *orf592* in Table S4).

Except for the ORFs that are homologous among ciliates, there are also some taxon or group-specific ORFs. For example, no homologous sequence was detected in other ciliate mitogenomes for the *orf_s1–s7* in the two *Strombidium* mitogenomes or for the six ORFs in the two *Euplotes* mitogenomes based on BLASTP searches (Figure 1, Table S4). *Orf192* is only detected in the mitogenomes of *H. grandinella* and *P. cristata*, and *orf549* is only detected in *H. grandinella*, *O. trifallax*, and *S. lemnae*. These taxon or group-specific ORFs might represent novel mitochondrial proteins or perform similar functions but be too divergent to find homologies [33,39].

### 3.3. Repeat Regions in Mitogenomes

In ciliate mitogenomes, there are three kinds of repeat regions: terminal inverted repeats (TIR) (repeat sequences with opposite directions at each end of the linear molecule), telomeric repeats (a telomeric sequence composed of repeat unit that is usually repeated several times), and central repeats (a sequence comprised of repeat units at approximately a central position of the linear molecule), though some mitogenomes may contain one, two, or all of them. TIR is a common characteristic of linear mitogenomes from diverse eukaryotes as reviewed in [39] and is proposed as a solution of the incomplete 5′ end replication problem for linear molecules [56]. For ciliate mitogenomes, the TIR is only detected in several ciliate species, including *Tetrahymena* species, *O. trifallax*, *S. lemnae*, and *L. strenua*, with the size ranging from ~1800–3100 bp (Table 2). Notably, the TIR region in *O. trifallax* mitogenome appears to be largely comprised of non-annotated ORFs, while the TIR in *S. lemnae’s* and *L. strenua’s* is largely comprised of the large subunit ribosomal RNAs and tRNAs, which is similar to that in *Tetrahymena* species. Coincidentally, a high frequency of structural rearrangement events is also observed within the mitogenomes of Spirotrichea, i.e., the existence of similar gene sets in both ends of the mitogenomes in *O. trifallax*, *S. lemnae*, and *L. strenua* (Figure 3). These suggest that, instead of as a solution of the 5′ end replication problem, the TIR in Spirotrichea mitogenomes may play a role in its structural rearrangements, which may result from terminal inverted gene duplications and subsequent successive gene losses through homologous recombination between both termini.

According to Nosek and Tomáška [56], we speculate that the telomeric repeats in the Spirotrichea mitogenomes may play important roles in solving the end replication problem. Most Spirotrichea mitogenomes are capped by telomeric repeats, with the same sequence at both ends and an unknown repeat number (Table 2). The undetected telomeric repeat in some Spirotrichea mitogenomes is probably due to the incompleteness of the mitogenomes. However, compared to the highly conserved telomeric sequences in the nuclear genome, the telomeric repeats in the Spirotrichea mitogenomes are highly variable, ranging from 13–36 bp, even among closely related species (e.g., two *Strombidium* species). The telomeric repeats are also found in *Tetrahymena* mitogenomes (31–53 bp), but even different at each end in the *T. pigmentosa* mitogenome [28]. It was proposed that mitochondrial telomeres are derived from mobile elements (transposons or plasmids) [56]. A linear mitochondrial plasmid with the 3′ end containing the same telomeric repeats as the primary mitogenome was found in *O. trifallax*, which supports the previous proposal of the telomere transfer between mitogenomes and linear plasmids [39].

An AT-rich central tandem repeat is discovered in all the available Spirotrichea mitogenomes, where a bidirectional transcription initiates from this region. The repeat region comprises a palindromic sequence and could be predicted to form stable stem-loop structures (Figure 1, Figure 3 and Figure 4). Similarly, *Tetrahymena* mitogenomes (Cl.: Oligohymenophorea) also contain a conserved putative GC-box control region located in the highly AT-rich stretch, which is speculated to be significant to bidirectional transcription [28]. Except for the ciliated protists, tandem repeat and stem-loop structures are also reported in mitogenomes of numerous organisms, for instance, unicellular cryptophyte algae (*Rhodomonas salina* [57] and *Hemiselmis andersenii* [58]), plants (*Chondrus crispus* [59] and *Porphyra purpurea* [60]) and animals (chickens [61] and lagomorphs [62]). According to previous studies, the tandem repeat and hairpin structure may be associated with a promoter and involved in the regulation of transcription initiation [57,58,61]. Additionally, the transcription control region of mitogenomes was demonstrated to be AT-rich [28,61]. All of these points mentioned above imply that the central tandem repeat may play an important role in the bidirectional transcription of the mitochondrial genes in Spirotrichea species.

Like the mitogenomes of diverse organisms [28,30,57,58], the position of transcription initiation also corresponds to origin of DNA replication, which means that the central repeat regions in the Spirotrichea mitogenomes could also be the replication origin. In *Paramecium* mitogenomes, the possession of covalently-closed single-stranded hairpins (consisting of direct tandem repeats) at one end and an open structure at the opposite side was found. The hairpin structure was demonstrated to be associated with a replication origin [27,63]. In these structural features, replication is initiated through the hairpin loop generating a dimer molecule, which is then processed to two cross-linked monomers [27,56]. Recently, more *Paramecium* mitogenomes were published [33]. We found that, instead of an open structure at the opposite end, a linear molecule terminates with telomeric repeats ranging from 23–33 bp. For the Spirotrichea mitogenomes, if we fold them from the central regions, their structures are surprisingly analogous to that in *Paramecium* mitogenomes: with covalently-closed sun-shape central repeat at one end and telomeric repeats at the other end (Figure 4K). As a consequence, we speculate that the central repeat region may serve as the replication initiation in Spirotrichea mitogenomes. More molecular information and experimental evidence are needed to test this speculation.

### 3.4. Phylogenetic Position of Halteria grandinella

The family Halteriidae, represented by *H. grandinella*, was assigned into the subclass of Oligotrichia in consideration of its similar morphological characteristics with oligotrichs, for example, the globular/ovoid cell shape, planktonic life style, the reduced somatic ciliature, apical oral membranelles, de novo-derived undulating membrane, and enantiotropy pattern for cell division [46,47]. All these characteristics are divergent with those in hypotrichs, such as mostly dorsoventrally compressed, mostly benthic life style, well-developed compound ventral cirri and dorsal kineties, oral membranelles on left-anterior portion of the ventral surface, as well as a synclastic pattern for cell division [48]. However, *H. grandinella* was always closely related to hypotrichs based on phylogenetic trees inferred from nuclear data [19,48,49,50,51,52]. This is concordant with our phylogenetic analyses based on mitochondrial data (Figure 7).

Except for the phylogenetic analyses, the characteristics of the mitogenome also support the close relationship of *H. grandinella* and hypotrichs. In the present study, the mitogenome of *H. grandinella* exhibits more extensive synteny with those of hypotrichs than oligotrichs, especially the complete collinearity with that of *O. trifallax* (Figure 3). Furthermore, the *ccmf* of *H. grandinella* is split into two parts, which is consistent with hypotrichs but different from the two oligotrichs whose *ccmf* is non-split (Figure 5). It is noteworthy that the mitogenomes of *Strombidium* species contain specific ORFs (*orf_s1–s7*) which are not detected in *H. grandinella*. The foregoing findings demonstrate that *Halteria* has a closer relationship with hypotrichs than oligotrichs.

According to Lynn and Kolisko [19] and Wang et al. 2019 [48], the morphological features shared between halteriids and oligotrichs are likely convergent characteristics for planktonic lifestyle and *H. grandinella* might be a “oligotrich-like” hypotrich. Both our mitogenome information and phylogenetic analyses support this hypothesis, though limited mitochondrial data in the subclass Oligotrichia is available now. Besides, as discussed in Wang et al. 2019 [48], *H. grandinella* shared more nuclear orthologous genes with oxytrichids than with oligotrichs. Considering that *Halteria* is the type genus of the family Halteriidae, we agree with Lynn [64] that Halteriidae should be classified into the subclass Hypotrichia and it has a closer relationship with the family Oxytrichidae than other families within Hypotrichia.

## 4. Materials and Methods

### 4.1. Cell Cultures

*Halteria grandinella* was collected from the pond of the Baihuayuan Park in Qingdao (36°04′ N, 120°22′ E), China. Several cells were isolated by glass micropipette under microscope and washed 4–5 times, and then were cultured in filtered and autoclaved in situ water at room temperature, with *Escherichia coli* as food source. *Strombidium* sp. was kindly supplied by Dr. Sheng-Fang Tsai of the Institute of Environmental Biology and Fisheries Science in National Taiwan Ocean University. *Strombidium* cf. *sulcatum* was sampled from Daya Bay in Guangdong (22°37′ N, 114°38′ E), China. Both *Strombidium* species were cultured in filtered and autoclaved marine water at room temperature with *E. coli* as food source. All of the three morphospecies were identified by live microscopic observation and silver impregnations [65].

### 4.2. Mitochondrial DNA, Genomic DNA Extraction, and High-Throughput Sequencing

For the cultures of *H. grandinella* and *Strombidium* sp., cells were starved and treated with antibiotic-antimycotic (2 mL for 1 L, ThermoFisher Scientific, Waltham, MA, USA; Cat No. 15,240,062) for about 24 h before mitochondria isolation. Samples were collected by centrifugation at 200× *g* for 3 min. The total numbers of cells were approximately 3 × 10^4^ for *H. grandinella* and 5 × 10^4^ for *Strombidium* sp. Mitochondria were isolated using a Qproteome Mitochondria Isolation Kit (Qiagen, Hilden, Germany; Cat No. 37,612), following the manufacturer’s protocol without washing with 0.9% sodium chloride solution. Subsequently, the DNA extraction was performed by DNeasy Blood & Tissue Kit (Qiagen, Hilden, Germany; Cat No. 69,506). Finally, the mitochondrial DNA was amplified using a REPLI-g^®^ Mitochondrial DNA Kit (Qiagen, Hilden, Germany; Cat No. 151,023) with 10 µL template DNA.

For the culture of *S.* cf. *sulcatum*, cells were starved and treated with the antibiotic-antimycotic as described above. After that, cells were harvested by filtering through a 5 µm membrane (Shanghai Xin Ya Purification Equipment, Shanghai, China). Subsequently, cells (ca. 2 × 10^5^) were lysed using Proteinase K solution from DNeasy Blood & Tissue Kit (Qiagen, Hilden, Germany; Cat No. 69,506) and a lysis buffer (10 mM Tris-HCl, 25 mM EDTA, 0.5% SDS) at 56 °C in a water bath for 3 h. Genomic DNA was then extracted by Phenol-Chloroform method. RNA was eliminated with RNase A (Omega Bio-tek, Norcross, GA, USA; Lot No. L10RG).

Three illumina libraries (350 bp) were constructed using NEBNext^®^ Ultra™ DNA Library Prep Kit (NEB, Ipswich, MA, USA; Cat No. E7370L) according to the manufacturer’s instructions: mitochondrial DNA of *H. grandinella*, mitochondrial DNA of *Strombidium* sp., and genomic DNA of *S.* cf. *sulcatum*. Paired-end sequencing (150 bp reads length) was performed using an Illumina HiSeq4000 sequencer (Novogene, Beijing, China). Sequencing adapters and low-quality reads (more than 50% bases with Q value = < 5) were removed by FASTX-Toolkit with the parameters of -q 5 -p 0.5 [66].

### 4.3. Mitogenome Assembly and Annotation

Mitogenomes of *H. grandinella* and *Strombidium* sp. as well as the whole genome of *S.* cf. *sulcatum*, were assembled using SPAdes v.3.11 with default parameters varying k-values: 21, 33, 55, and 77 [13,67]. Afterwards, assemblies were screened by BLASTN (e-value = < 1 × 10^−5^) using eight mitogenomes downloaded from the National Center for Biotechnology Information (NCBI) as a reference database, including *Oxytricha trifallax* (acc. no. JN383843), *Tetrahymena pyriformis* (acc. no. AF160864), *Tetrahymena thermophila* (acc. no. AF396436), *Paramecium aurelia* (acc. no. NC001324), *Paramecium caudatum* (acc. no. FN424190), *Euplotes minuta* (acc. no. GQ903130), *Euplotes crassus* (acc. no. GQ903131), and *Nyctotherus ovalis* (acc. no. GU057832).

For *H. grandinella* and *Strombidium* sp., one resulting contig (i.e., mitogenome) possessing high similarity with the reference data was found in each species (similarity: >72.24% in *H. grandinella*; >72.73% in *Strombidium* sp.). For *S.* cf. *sulcatum*, three contigs were recovered (similarity: >72.82%). These three contigs were subsequently used as references for the second search in the genomic data with BLASTN (using an e-value threshold of = < 1 × 10^−5^) and another six contigs were obtained. Nine mitochondrial contigs of *S.* cf. *sulcatum* with sizes ranging from about 1.2 kb to 19 kb were assembled into two scaffolds by SeqMan v.7.1.0 (DNAStar). Polymerase chain reactions (PCRs) were performed to confirm the junctions of these contigs and link the two scaffolds together into a linear mitogenome (Figure S1). The PCR amplifications were performed using the 2× EasyTaq^®^ PCR SuperMix (Transgen Biotech, Beijing, China; Cat No. AS111–14), with primers designed according to the nine mitochondrial contigs (Table S1). PCR products were purified by EasyPure Quick Gel Extraction Kit (Transgen Biotech, Beijing, China; Cat No. EG101–01) and cloned using a pEASY-T1 Cloning Kit (Transgen Biotech, Beijing, China; Cat No. CT101–01), or directly sequenced bidirectionally in Tsingke Biological Technology Company (Beijing, China). Raw reads data of the three taxa were respectively remapped onto the mitogenome to calculate the coverage through HISAT2 v.2.1.0 [68] and SAMtools [69]. Integrative Genomics Viewer v.2.4.6 [70] was employed to visualize coverage results (Figure S2).

Three mitogenomes were annotated firstly using both MITOS and MFannot with genetic code 4 of “the mold, protozoan, and coelenterate mitochondrial code and the mycoplasma/spiroplasma code” [71,72]. Then, the protein-coding genes were further verified by the NCBI Open Reading Frame Finder (https://www.ncbi.nlm.nih.gov/orffinder (accessed on 23 February 2021)) and annotated by searching against the NCBI non-redundant protein sequences (nr) database with SmartBLAST or BLASTP. The ribosomal RNA (rRNA) genes were confirmed by BLASTN searches against the NCBI nucleotide collection (nr/nt) database. The transfer RNA (tRNA) genes were also predicted using tRNAscan-SE v.2.0 with default mode and other mitogenomes as reference sources [73]. Mitogenome maps were drawn using OGDRAW [74]. The secondary structures of the central repeated portion of the mitogenomes were predicted using the Mfold web server (http://www.unafold.org/mfold/applications/rna-folding-form.php (accessed on 23 February 2021)) with default parameters. RnaViz v.2.0 [75] was used for aesthetic purposes. The transmembrane (TM) structure of the ccmf protein which is involved in cytochrome c maturation in mitochondria was predicted by TMHMM v.2.0 [76].

### 4.4. Phylogenetic Analyses and Topology Testing

Fourteen conserved mitochondrial protein sequences were used to construct phylogenetic trees. Newly characterized sequences were combined with relevant orthologs of the other 31 ciliates downloaded from the GenBank database, resulting in fourteen mitochondrial datasets (Table S2). *Stentor coeruleus* and *Gruberia lanceolata* were selected as the outgroup taxa. The fourteen datasets were aligned using the MAFFT algorithm on the GUIDANCE2 Server (http://guidance.tau.ac.il/ver2/ (accessed on 23 February 2021)) and trimmed by trimAl v.1.4 with default parameters [77]. Subsequently, fourteen mitochondrial alignments were concatenated using SeaView v.4 [78]. The final alignments used for phylogenetic analyses included 3730 sites. Maximum likelihood (ML) analysis was performed by IQ-tree v.1.5.5 [79] with the model LG+C60+F+G4. In total, 100 bootstrap replicates were run to calculate the standard nonparametric bootstrap supports. Bayesian inference (BI) analysis was carried out by PhyloBayes v.4.1 [80] under the model CAT+GTR. Four Markov chain Monte Carlo (MCMC) simulations were run for 1,000,000 generations. The bpcomp program of PhyloBayes was performed to compare the discrepancy of bipartition frequencies, generating a consensus tree. The tree topologies were visualized using FigTree v.1.4.4 (http://tree.bio.ed.ac.uk/software/figtree/ (accessed on 23 February 2021)).

To test the monophyly of *H. grandinella* and the subclass Oligotrichia, the constrained ML tree was generated with a constraint block by enforcing the monophyly of *H. grandinella* and two *Strombidium* species. Subsequently, the approximately unbiased (AU) test [81] was performed using IQ-tree v.1.5.5 [79].

## Figures and Tables

**Table 1:** Main features of all the available mitogenomes of aerobic ciliates and a representative hydrogenosome genome of anaerobic ciliates. Newly characterized mitogenomes are in bold.

Species	Accession No.	Genome Size (bp)	Gene Region (bp)	Intergenic Region (bp)	Overall A+T Content (%)	Known Protein Genes	tRNA Genes	rRNA Genes
***Strombidium* sp.**	**MT471315**	51,232	48,614	2618	77.32	29	8	2
***Strombidium* cf. *sulcatum***	**MT471316**	54,912	52,218	2694	71.68	29	9	2
***Halteria grandinella***	**MT471317**	50,085	45,401	4684	80.20	29	9	2
*Oxytricha trifallax*	JN383843	69,800	61,685	8115	76.17	29	11	2
*Stylonychia lemnae*	KX524144	67,745	51,501	16,244	74.05	29	12	3
*Laurentiella strenua*	KX529838	66,721	48,266	18,455	75.75	30	7	3
*Paraurostyla* sp.	KX524143	65,186	42,149	23,037	80.59	29	8	2
*Urostyla grandis*	KX494929	60,924	37,327	23,597	61.12	27	7	2
*Pseudourostyla cristata*	MH888186	76,660	59,952	16,708	78.27	29	9	2
*Euplotes minuta*	GQ903130	41,978	40,257	1721	64.74	20	6	2
*Euplotes crassus*	GQ903131	33,688	32,273	1415	65.61	17	5	2
*Tetrahymena pyriformis*	AF160864	47,296	45,285	2011	78.68	24	8	3
*Tetrahymena thermophila*	AF396436	47,577	45,619	1958	79.24	21	8	3
*Tetrahymena malaccensis*	DQ927303	47,691	45,528	2163	80.10	21	8	3
*Tetrahymena paravorax*	DQ927304	47,496	44,812	2684	81.51	21	8	3
*Tetrahymena pigmentosa*	DQ927305	46,990	44,889	2101	81.46	21	8	3
*Tetrahymena rostrata*	MN025427	47,235	45,336	1899	78.23	23	8	3
*Ichthyophthirius multifiliis*	JN227086	51,686	43,469	8217	83.62	21	5	3
*Uronema marinum*	MG272262	39,845	35,544	4301	81.00	24	6	2
*Paramecium caudatum*	FN424190	43,660	41,091	2569	77.62	24	3	2
*Paramecium aurelia*	NC001324	40,469	26,808	13,661	58.76	20	4	2
*Paramecium tetraurelia*	-	40,267	39,204	1063	58.34	23	3	2
*Paramecium sexaurelia*	-	40,015	37,474	2541	60.24	24	3	2
*Paramecium multimicronucleatum*	-	39,460	33,539	5921	80.38	21	34	2
*Paramecium biaurelia*	-	39,870	36,297	3573	59.89	24	3	2
*Paramecium octaurelia*	-	39,850	37,784	2066	59.66	23	3	2
*Paramecium novaurelia*	-	59,002	36,996	22,006	65.02	23	3	2
*Paramecium decaurelia*	-	42,742	37,779	4963	58.64	24	3	2
*Paramecium dodecaurelia*	-	40,335	37,947	2388	58.52	23	3	2
*Paramecium quadecaurelia*	-	41,844	37,450	4394	58.75	23	3	2
*Paramecium jenningsi*	-	40,161	38,010	2151	59.44	24	3	2
*Nyctotherus ovalis*	GU057832	41,666	32,511	9155	58.52	16	3	2
*Stentor coeruleus*	MPUH01000652	41,645	36,154	5491	80.15	22	5	2
*Gruberia lanceolata*	MK301177	39,988	30,910	9078	79.58	25	4	2

- indicates the mitogenome data of ten *Paramecium* species which are not available on the NCBI database but can be accessed on Zenodo (https://doi.org/10.5281/zenodo.2539699 (accessed on 23 February 2021)).

**Table 2:** Information of mitogenome repeats within the class Spirotrichea. * Denotes absence.

Species	Central Repeat			Telomeric Repeat (5′–3′)	Terminal Inverted Repeat
	Length (bp)	A+T Content	Repeat Unit (Number of Repeats)		
*Strombidium* sp.	170	100.00%	ATAATATAATAATAT (11)	CTCCCTTATCTAGTCTTT (both ends)	*
*Strombidium* cf. *sulcatum*	142	96.48%	ATAAATTTAATTTTA (2) + irregular sequence for the rest	TTATATCCTTTCTCCCCTATATCTCTATAGTACT (both ends)	*
*Halteria grandinella*	168	94.05%	TATACATATAATATATA (9)	AAAACAGCTCCGTTCCAATACTACTAACTAA (both ends)	*
*Oxytricha trifallax*	~285	96.76%	TATATAAA (11) + TATAAATAAA (3) + AAAAAG (5)	CGACTCCTCTATCCTCATCCTAGACTCCGCTTACT (both ends)	~1800 bp
*Stylonychia lemnae*	~607	92.29%	TATARTAGTTATATTATA (27)	TTCATACCTTTACTAGATACCCGCCTCCGGCTCTCC (3′ end)	~3100 bp
*Laurentiella strenua*	733	98.91%	ATATAAATGTATATAA (7) + ATAAA(TA)_n_T (49) + TTT(AT)_n_ (4), *n* = 0–8	CCTACTACGCTTCATACGCTAAA (partial) (both ends)	~2400 bp
*Paraurostyla* sp.	~802	98.86%	ATATAACAAATA (7) + AAATAA(TA)_n_AT (20), *n* = 2–29	*	*
*Urostyla grandis*	~279	95.91%	ATATATTTATTAATATATAGTAT (10)	GTAGCACATGTAG (3′ end)	*
*Pseudourostyla cristata*	80	86.25%	TATATATACATATAC (3) + (TA)_n_C (3), *n* = 3 or 5	*	*
*Euplotes minuta*	~1596	83.36%	ATAGTATATAATGTATAC (63) + ATAGTATATAATGTTAC (1) + ATAGTATATAATTGTTAC (18)	*	*
*Euplotes crassus*	~416	83.54%	ATAGTATATAATGTATAC (15)	*	*

## Data Availability

Three new mitogenome assemblies and annotation data in this study have been submitted to the GenBank database under accession numbers as follows: MT471315 for mitogenome of *Strombidium* sp., MT471316 for mitogenome of *Strombidium* cf. *sulcatum*, and MT471317 for mitogenome of *Halteria grandinella*.

## Supplementary Materials

The following are available online at https://www.mdpi.com/1422-0067/22/5/2546/s1.
