# Supplementary material for: Characterization and Comparative Analyses of Mitochondrial Genomes in Single-Celled Eukaryotes to Shed Light on the Diversity and Evolution of Linear Molecular Architecture
Source: Int J Mol Sci. 2021 Mar 3;22(5):2546. doi: 10.3390/ijms22052546 (PMC7961746; doi:10.3390/ijms22052546)
Supplement: Supplementary file 1 [file ijms-22-02546-s001.pdf]

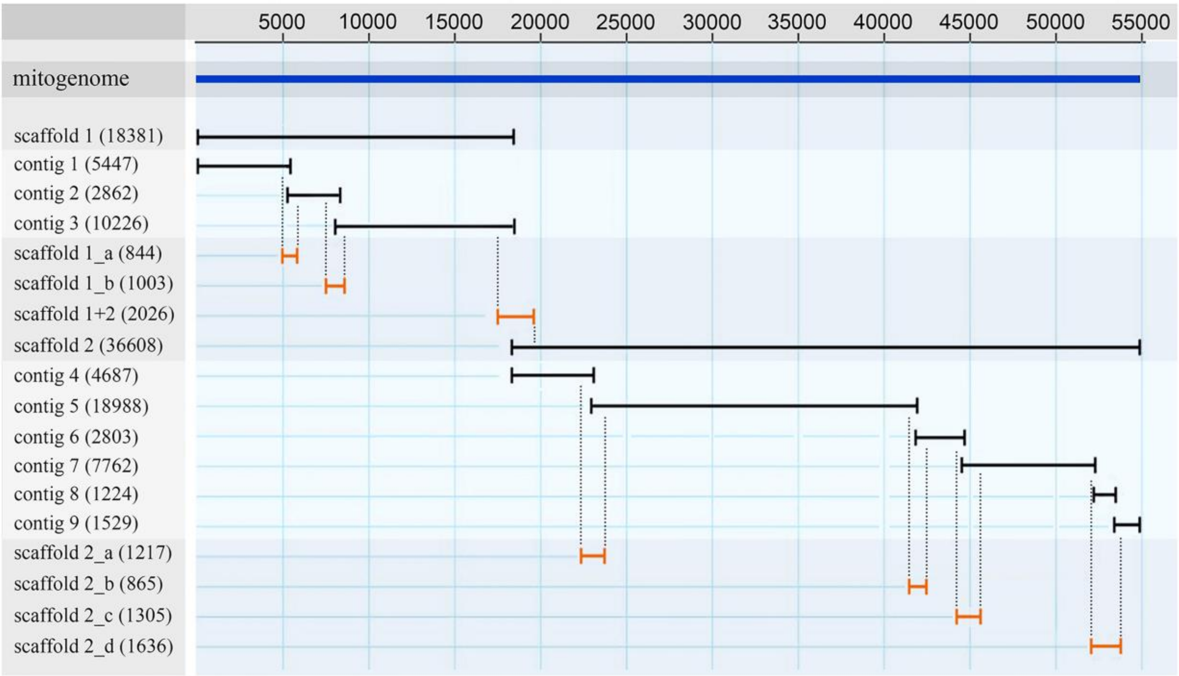

Figure S1. Schematic maps demonstrating how whole mitogenome was confirmed in *Strombidium* cf. *sulcatum*. Black lines indicate the mitochondrial contigs recovered from genomic assembly of *Strombidium* cf. *sulcatum*. Red lines represent PCR results used to link mitochondrial contigs. The vertical dashed lines indicate the binding positions of PCR products. Numbers in parentheses suggest the lengths (bp) of the fragments.

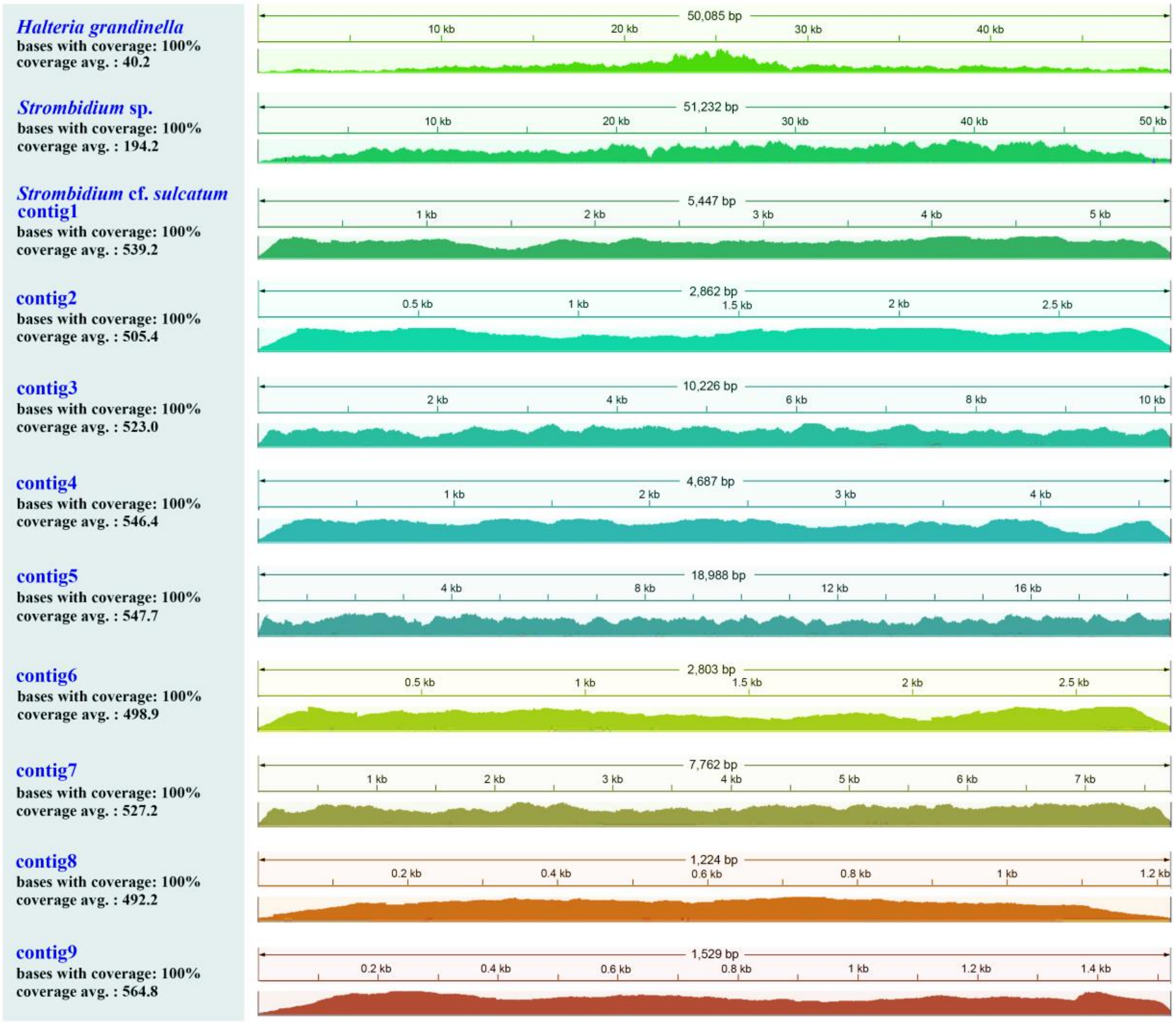

Figure S2. Coverage plots for mitochondrial contigs of *Halteria grandinella*, *Strombidium* sp. and *Strombidium* cf. *sulcatum*. The horizontal and vertical axes indicate contig length and coverage, respectively.

Table 1. Primers for PCR amplification to confirm mitogenome of *Strombidium* cf. *sulcatum*.

PCR fragments	Primers	Sequences (5′–3′)
Scaffold 1_a	413_F1	CAGTAGCCAAGAATATAGCG
	1340_R1	ACTTCTAACTGAGTAGCATC
Scaffold 1_b	423_F1	CAAGACCTATCCATATCTCCCC
	1514_R2	GCCTTTTGTAGCAAGCCCATCA
Scaffold 2_a	195_F1	ATTTACAGTCTGCCGCTTTCG
	1518_R1	TGACTTTGGTAAGGCGTAGG
Scaffold 2_b	295_F1	GACTTTGAGCAGGTTGATACC
	1250_R1	TACTTCTTCGTAGTCGGGAG
Scaffold 2_c	503_F1	TAAGTATTCGATTATCCGCAGG
	1926_R1	ACCCGATTACAGAGGTTACG
Scaffold 2_d	1033_F3	CTGCCACATTTCAAGTAGAC
	2668_R2	ATGGAATCCCTTATCCTGAA
Scaffold 1+2	51_F1	ACGATAGGGAGTGAATACGG
	1488_R1	ACTCGCCTGTATCAAGTCGG

Table S2. GenBank accession numbers of mitochondrial protein sequences used for the phylogenetic trees. Newly characterized sequences are in bold.

Species	nad4	nad4L	nad5	nad7	nad9	nad10	cob	cox1	cox2	rps3	rps12	rpl2	rpl14	rpl16
Strombidium sp.	**XXX**	**XXX**	**XXX**	**XXX**	**XXX**	**XXX**	**XXX**	**XXX**	**XXX**	**XXX**	**XXX**	**XXX**	**XXX**	**XXX**
Strombidium cf. *sulcatum*	**XXX**	**XXX**	**XXX**	**XXX**	**XXX**	**XXX**	**XXX**	**XXX**	**XXX**	**XXX**	**XXX**	**XXX**	**XXX**	**XXX**
Halteria grandinella	**XXX**	**XXX**	**XXX**	**XXX**	**XXX**	**XXX**	**XXX**	**XXX**	**XXX**	**XXX**	**XXX**	**XXX**	**XXX**	**XXX**
Oxytricha trifallax	AEV66679	AEV66697	AEV66639	AEV66667	AEV66686	AEV66677	AEV66649	AEV66698	AEV66701	AEV66694	AEV66678	AEV66675	AEV66700	AEV66681
Stylonychia lemnae	ASY95723	ASY95740	KX524144	ASY95733	ASY95748	ASY95752	ASY95741	ASY95744	ASY95747	KX524144	ASY95728	ASY95727	ASY95731	ASY95746
Laurentiella strenua	APW82405	APW82422	APW82424	APW82402	APW82406	APW82435	APW82441	APW82411	APW82428	APW82425	APW82431	APW82423	APW82412	APW82420
Paraurostyla sp.	ASY95711	ASY95717	ASY95710	ASY95698	ASY95690	ASY95719	ASY95713	KX524143	ASY95718	KX524143	ASY95704	ASY95706	ASY95700	ASY95694
Urostyla grandis	APW82389	APW82397	APW82373	APW82388	APW82396	APW82372	APW82384	APW82381	APW82392	APW82394	APW82386	APW82380	APW82382	APW82385
Pseudourostyla cristata	QCU82614	QCU82604	QCU82636	QCU82622	QCU82609	QCU82616	QCU82631	QCU82603	QCU82601	QCU82607	QCU82615	QCU82618	QCU82602	QCU82613
Euplotes minuta	ACX30958	ACX30950	ACX30940	ACX30966	ACX30954	ACX30960	ACX30945	ACX30949	ACX30947	ACX30952	ACX30959	ACX30962	ACX30948	ACX30957
Euplotes crassus	ACX30996	ACX30985	ACX30976	ACX31004	ACX30990	ACX30998	ACX30980	ACX30984	ACX30982	ACX30988	ACX30997	ACX31000	ACX30983	ACX30995
Tetrahymena pyriformis	AAD41955	AAD41940	AAD41944	AAD41924	AAD41941	AAD41921	AAD41943	AAD41950	AAD41945	AAD41917	AAD41922	AAD41919	AAD41953	AAD41935
Tetrahymena thermophila	AAK77603	AAK77588	AAK77593	AAK77572	AAK77589	AAK77569	AAK77592	AAK77599	AAK77594	AAK77565	AAK77570	AAK77567	AAK77602	AAK77583
Tetrahymena malaccensis	ABI51668	ABI51652	ABI51657	ABI51636	ABI51653	ABI51633	ABI51656	ABI51663	ABI51658	ABI51629	ABI51634	ABI51631	ABI51666	ABI51647
Tetrahymena paravorax	ABI51712	ABI51697	ABI51701	ABI51681	ABI51698	ABI51678	ABI51700	ABI51707	ABI51702	ABI51674	ABI51679	ABI51676	ABI51710	ABI51692
Tetrahymena pigmentosa	ABI51756	ABI51741	ABI51745	ABI51725	ABI51742	ABI51721	ABI51744	ABI51751	ABI51746	ABI51718	ABI51722	ABI51720	ABI51754	ABI51736
Tetrahymena rostrata	QGS65290	QGS65275	QGS65279	QGS65259	QGS65276	QGS65256	QGS65278	QGS65285	QGS65280	QGS65252	QGS65257	QGS65254	QGS65288	QGS65270
Ichthyophthirius multifiliis	AEL89289	AEL89274	AEL89279	AEL89261	AEL89275	AEL89258	AEL89278	AEL89284	AEL89280	AEL89254	AEL89259	AEL89256	AEL89287	AEL89270
Uronema marinum	AXJ93331	AXJ93357	AXJ93360	AXJ93343	AXJ93358	AXJ93340	AXJ93359	AXJ93326	AXJ93361	AXJ93336	AXJ93341	AXJ93338	AXJ93329	AXJ93353
Paramecium caudatum	CAZ66798	CAZ66836	CAZ66808	CAZ66819	CAZ66837	CAZ66816	CAZ66809	CAZ66803	CAZ66807	CAZ66812	CAZ66817	CAZ66814	CAZ66800	CAZ66832
Paramecium aurelia	NP_039496	NP_039468	NP_039486	NP_039475	NP_039467	NP_039478	NP_039485	NP_039493	NP_039488	NP_039481	NP_039477	NP_039480	NP_039495	**
Paramecium tetraurelia	-	-	-	-	-	-	-	-	-	-	-	-	-	-
Paramecium sexaurelia	-	-	-	-	-	-	-	-	-	-	-	-	-	-
Paramecium multimicronucleatum	-	-	-	-	-	-	-	-	-	-	-	-	-	-
Paramecium biaurelia	-	-	-	-	-	-	-	-	-	-	-	-	-	-

Paramecium octaurelia	-	-	-	-	-	-	-	-	-	-	-	-	-	-
Paramecium novaurelia	-	-	-	-	-	-	-	-	-	-	-	-	-	-
Paramecium decaurelia	-	-	-	-	-	-	-	-	-	-	-	-	-	-
Paramecium dodecaurelia	-	-	-	-	-	-	-	-	-	-	-	-	-	-
Paramecium quadecaurelia	-	-	-	-	-	-	-	-	-	-	-	-	-	-
Paramecium jenningsi	-	-	-	-	-	-	-	-	-	-	-	-	-	-
Nyctotherus ovalis	ADN85877	ADN85881	ADN85887	ADN85892	ADN85869	ADN85879	*	*	*	*	ADN85897	ADN85890	ADN85884	**
Stentor coeruleus	**	**	**	**	**	**	**	**	**	*	**	**	**	*
Gruberia lanceolata	QIB71983	QIB71970	QIB71966	QIB71988	QIB71977	QIB71976	QIB71982	QIB71965	QIB71967	QIB71978	QIB71972	QIB71980	QIB71996	QIB71993

Note: - indicates the mitogenome data of ten *Paramecium* species are not available on NCBI database but can be accessed on Zenodo (https://doi.org/10.5281/zenodo.2539699); * indicates absent; ** indicates mitochondrial proteins of *Stentor coeruleus* (acc. no. MPUH01000652) and rpl16 of *Paramecium aurelia* (acc. no. NC001324) and *Nyctotherus ovalis* (acc. no. GU057832) are not available on NCBI database but annotated in the present work based on mitogenome data.

Table S3. Overlapped mitochondrial genes of three newly sequenced species in the present work.

Species	Overlapped Genes	Overlapped Length (bp)
Strombidium sp.	*nad1_a + orf_s2*	14
	rps8 + tRNA_Gln	15
	rpl2 + orf_s4	10
	orf_s4 + nad10	25
	nad4 + rpl16	19
	rps10 + tRNA_Glu	42
	rps3_b + tRNA_Trp	46
Strombidium cf. *sulcatum*	*rps2 + nad5*	25
	rps8 + tRNA_Gln	43
	orf_s3 + rps14	7
	rps4 + rps13	62
	nad9 + tRNA_Phe	82
	orf578 + tRNA_Tyr	81
	rps3_b + tRNA_Trp	25
Halteria grandinella	*orf192 + ccmf_ii*	43
	orf535 + tRNA_His	73
	orf535 + cob	11
	cob + tRNA_His	4
	rps8 + tRNA_Gln	43
	rps14 + nad7	4
	rps4 + rps13	10
	rps13 + rps19	25
	rps19 + rpl2	4
	rpl2 + orf561	32
	orf561 + nad10	190
	rps10 + tRNA_Glu	58
	nad9 + tRNA_Phe	8
	rps3_b + tRNA_Trp	60
	nad4L + cox1	13

Table 4. ORFs with unknown function encoded in mitogenomes of the class Spirotrichea.

Group	ORFs	*Strombidium* sp.	*Strombidium* cf. *sulcatum*	*Halteria grandinella*	*Oxytricha trifallax*	*Stylonychia lemnae*	*Laurentiella strenua*	*Paraurostyla* sp.	*Urostyla grandis*	*Pseudourostyla cristata*	*Euplotes minuta*	*Euplotes crassus*
Homologous ORFs	*orf535*	+	+	+	+	*	+	*	*	*	*	*
	orf546	+	+	+	+	+	+	*	+	*orf393*	*orf380*	*
	orf578	+	+	+	+	+	+	+	*	*orf439*	*	*
	orf583	+	+	+	+	+	+	+	+	*orf373*	*orf268*	*orf311*
	orf592	+	+	+	+	+	+	+	*	*orf262*	*orf78*	*orf129*
	orf192	*	*	+	*	*	*	*	*	+	*	*
	orf549	*	*	+	+	+	*	*	*	*	*	*
	orf561	*	*	+	+	+	+	+	*	*orf256*	*orf267*	*orf297*
	orf584	*	*	+	+	+	+	+	*	*orf178*	*	*
	orf547	*	*	*	+	*	*	+	+	*	*	*
	orf_s1	+	+	*	*	*	*	*	*	*	*	*
	orf_s3	+	+	*	*	*	*	*	*	*	*	*
	orf_s4	+	+	*	*	*	*	*	*	*	*	*
	orf_s5	+	+	*	*	*	*	*	*	*	*	*
	orf_s6	+	+	*	*	*	*	*	*	*	*	*
	orf_s7	+	+	*	*	*	*	*	*	*	*	*
	orf259	*	*	*	*	*	*	*	*	*	+	*orf449*
	orf163	*	*	*	*	*	*	*	*	*	+	*orf197*
	orf187	*	*	*	*	*	*	*	*	*	+	*orf134*
	orf96	*	*	*	*	*	*	*	*	*	+	*orf101*
	orf111	*	*	*	*	*	*	*	*	*	+	*orf175*
	orf155	*	*	*	*	*	*	*	*	*	+	*orf156*
Non-homologous ORFs		1	0	0	50	0	0	0	0	14	6	3

Note: + denotes present; * denotes absent; numbers indicate the number of non-homologous ORFs. Homologous ORFs identified according to blastp searches and gene position on mitogenome are shown in the same row.
